# On Clasps

**Published:** 1858-06

**Authors:** C. W. Spalding


### ON CLASPS.

BY C. W. SPALDING, D. D. S.

A very large and a very useful class of cases calling for the restoration of teeth, is that of partial sets, requiring some kind of attachment to the remaining natural organs.

It is well known that the employment of the broad metallic bands commonly used in clasping these natural organs is in most cases followed, sooner or later, by the decay and final loss of the teeth embraced. This evil has long been recognized as a very serious one, and various expedients have been resorted to, with a view to rid our practice altogether of the use of clasps in any form. These attempts have been attended by partial success, and many practitioners now dispense with clasps in a certain class of cases where they were formerly deemed indispensable. Clasps are, however, still extensively used, and will no doubt continue to be, in spite of the injurious effects which their use is known to produce, and which have long stood in formidable array against them.

The principal cause of the decay of teeth consequent upon their use, is the fact that they favor the accumulation of mucus and soft food between the clasp and the teeth. These substances are retained and protected by the shielding clasp, and often remain for a long time in contact with the surface of the tooth, producing those corrosive and mischievous effects which are so often met with in cases where clasps of the usual form have been worn for any considerable length of time.

I need not attempt to describe the *modus operandi* by which such well known results are produced; the simple suggestion of the agents at work is sufficient to bring to mind the manner of its accomplishment.

It seems important that some method should be devised to prevent this wholesale destruction of sound natural organs, and at the same time permit their use in sustaining artificial ones. The suggestions which follow are submitted with the view of contributing *somethiny* towards the accomplishment of such a result.

In the first place, then, the plate should be of liberal dimensions, covering sufficient surface to afford a broad base for the support of the teeth which are to be placed upon it, so that the plate itself shall give all the support possible to the teeth relying upon the clasps to simply retain the plate in *situ*. If the plate is ample and accurately fitted to the palatal arch, a small amount of force will in most cases be found requisite to retain it in position. The writer has for many years been in the habit of employing *narrow* clasps for this purpsse, making them of sufficient thickness to give the required strength, and attaching them to the plate by means of standards, so arranged as to induce the removal of accumulations between the clasp and tooth, by the circulation of the saliva.

The use of one or more standards as a means of attachment, also provides, by a variation of their length for the grasping of the tooth at any desired point. If the tooth is long, and particularly if it is at the same time bell-crowned, the point selected should be toward the grinding surface, and as far from the gum as is found practicable. If the tooth is short and of such form that it can be successfully clasped at no other point than that near the gum, the plate should be cut away at least one, or one and a half lines from the tooth, and standards introduced for the purpose of promoting circulation, by affording a free passage for the ingress and egress of fluids. These standards should also be *narrow*, no wider than the clasp itself, and should constitute the only point of union between clasp and plate. Half-round wire will be found to be a very convenient article for making clasps. The particular *form* of the clasp is, however, immaterial if it is both narrow and strong.

The initial point of decay under clasps is almost always near the margin of the gum, and the object in adopting narrow detached clasps is to leave that portion of the tooth uncovered, and consequently, subject, in a considerable degree, to the same action of the fluids of the mouth, as would take place if no clasps were present.

It is not claimed that the above method of clasping will always preserve the teeth from injury, but it is claimed that much less injury would result from the use of the clasps described than usually follows the use of the ordinary broad bands.

An opportunity recently presented itself for examining a case where narrow clasps have been worn some ten or twelve years, and the clasped teeth were found still unaffected by their use, and presented as healthy an appearance as any others in the mouth. The plate worn is of fair size, and supports seven teeth, including the four incisors.

No mention has here been made of the use of clasps upon the lower teeth for the reason that their use is believed to be entirely unnecessary, except in cases where *front* teeth *only* are to be supplied. The remarks on upper sets apply with equal force to this class of cases.—*Amer. Dental Review.*

